# Supplementary material for: The Genomic Complexity of Invasion: Cryptic Lineages, Founder Effects and Sex Determination in Armoured Catfish
Source: Mol Ecol. 2026 Oct 27;35(20):e70609. doi: 10.1111/mec.70609 (PMC13714160; doi:10.1111/mec.70609)

MOLECULAR ECOLOGY

Supplemental Information for:

The genomic complexity of invasion: cryptic lineages, founder effects, and sex determination in armored catfish

Wynne V. Radcliffe[1,2], Daemin Kim[1,2], Kiedon Bryant[2], Christopher L. Riggins[3], Thomas Heard[3], Monica McGarrity[5], Daniel J. Daugherty[6], Joshuah S. Perkin[4], Rachel L. Moran[1,2]

[1]Department of Ecology and Evolution, University of Chicago, Chicago, Illinois, USA
[2]Department of Biology, Texas A&M University, College Station, Texas, USA
[3]Meadows Center for Water and the Environment, Texas State University, San Marcos, Texas, USA
[4]Department of Ecology and Conservation Biology, Texas A&M University, College Station, Texas, USA
[5]Texas Parks and Wildlife Department, Inland Fisheries Division, Austin, Texas, USA
[6]Texas Parks and Wildlife Department, Heart of the Hills Fisheries Science Center, Mountain Home, Texas, USA

Table of Contents:

**Table 1: San Marcos female sequencing stats**	Pages 2-3
**Table 2: San Marcos male sequencing stats**	Pages 4-5
**Table 3: Comal female sequencing stats**	Pages 6-7
**Table 4: Comal male sequencing stats**	Pages 8-9
**Table 5: San Felipe female sequencing stats**	Pages 10-11
**Table 6: San Felipe male sequencing stats**	Pages 12-13
**Table 7: Repeat families**	Pages 14-16
**Table 8: COI sequence metadata**	Pages 17-46
**Table 9: CV error rates**	Page 47
**Figure 1: Full COI phylogeny**	Page 48
**Figure 2: ADMIXTURE plots for K=1 to K=5**	Page 49
**Figure 3: “True” *Hypostomus* PCA**	Page 50
**Figure 4: *Pterygoplichthys* k-mer density**	Page 51

MOLECULAR ECOLOGY

Supplementary Table 1: Sequencing statistics and quality for n = 11 San Marcos female samples at each stage of the data processing pipeline for raw reads as well as coverage throughout the alignment workflow. Coverage statistics are based on an alignment to *Hypostomus* reference genome.

	F1	F2	F3	F4	F5	F6	F7	F8	F9	F10	F11
**R1**											
Raw (Lane 1)	57270881	54601736	50423678	52117557	44115779	56806880	45036518	47645233	74228760	54108225	70935253
Raw (Lane 2)	57417123	54442246	49893146	51290629	44653061	56513183	44685820	47382193	73530669	53234847	70666892
Raw (Lane 3)	57292580	54557315	50801311	51935584	43250105	56790951	45252923	47171993	74142678	53742428	71631978
Raw Total	171980584	163601297	151118135	155343770	132018945	170111014	134975261	142199419	221902107	161085500	213234123
Concatenated	171980584	163601297	151118135	155343770	132018945	170111014	134975261	142199419	221902107	161085500	213234123
CutAdapt	171980584	163601297	151118135	155343770	132018945	170111014	134975261	142199419	221902107	161085500	213234123
Trimmomatic Out (R1)	153746590	143240504	133215320	137987946	120713082	150949144	119205556	127774380	199338553	141798864	189678708
**R2**											
Raw (Lane 1)	57270881	54601736	50423678	52117557	44115779	56806880	45036518	47645233	74228760	54108225	70935253
Raw (Lane 2)	57417123	54442246	49893146	51290629	44653061	56513183	44685820	47382193	73530669	53234847	70666892
Raw (Lane 3)	57292580	54557315	50801311	51935584	43250105	56790951	45252923	47171993	74142678	53742428	71631978
Raw Total	171980584	163601297	151118135	155343770	132018945	170111014	134975261	142199419	221902107	161085500	213234123
Concatenated	171980584	163601297	151118135	155343770	132018945	170111014	134975261	142199419	221902107	161085500	213234123
CutAdapt	171980584	163601297	151118135	155343770	132018945	170111014	134975261	142199419	221902107	161085500	213234123
Trimmomatic	153746590	143240504	133215320	137987946	120713082	150949144	119205556	127774380	199338553	141798864	189678708
**Total # Reads and Base Pairs**											
Total reads	169494559	160818662	148450428	152299520	130212374	167401005	132608474	139803052	217861776	158016197	209820673
Total base pairs	48486172350	45608874900	42249862200	43543119900	37638818400	47752522350	37772104500	40136614800	62580049350	44972259150	59924907150
**Coverage**											
Pre-alignment	24.243086175	22.80443745	21.1249311	21.77155995	18.8194092	23.876261175	18.88605225	20.0683074	31.290024675	22.486129575	29.962453575

Post-alignment	21.9566	20.3221	19.1833	19.7845	16.8709	21.5172	17.1483	18.2744	28.33	20.3378	27.043
Rgadd	21.9566	20.3221	19.1833	19.7845	16.8709	21.5172	17.1483	18.2744	28.33	20.3378	27.043
Dupsmarked	12.0193	12.9093	11.8424	12.6621	12.0495	13.7355	10.2776	12.1704	18.8262	13.0908	16.9319
Mapped	12.0193	12.9093	11.8424	12.6621	12.0495	13.7355	10.2776	12.1704	18.8262	13.0908	16.9319

MOLECULAR ECOLOGY

Supplementary Table 2: Sequencing statistics and quality for n = 11 San Marcos male samples at each stage of the data processing pipeline for raw reads as well as coverage throughout the alignment workflow. Coverage statistics are based on an alignment to *Hypostomus* reference genome. Missing data represented with "NA".

	M1	M2	M4	M5	M6	M7	M8	M9	M10	M11	M12
**R1**											
Raw (Lane 1)	45998565	57989603	57818558	111679861	48846257	55013819	104812053	64538526	44939362	56849778	45457247
Raw (Lane 2)	45447440	57362011	57875957	110669912	48737863	54564082	103793190	63803646	45268387	55930652	45420521
Raw (Lane 3)	46177093	57466765	57978466	110704549	48578805	54617398	105402722	64314228	45231788	56584564	45310625
Raw Total	137623098	172818379	173672981	333054322	146162925	164195299	314007965	192656400	135439537	169364994	136188393
Concatenated	137623098	172818379	173672981	NA	146162925	164195299	NA	192656400	135439537	169364994	136188393
CutAdapt	137623098	172818379	173672981	333054322	146162925	164195299	314007965	192656400	135439537	169364994	136188393
Trimmomatic	123570922	155399646	158099558	301999780	133061301	147385367	284519156	172659814	121788673	150528728	118981648
**R2**											
Raw (Lane 1)	45998565	57989603	57818558	111679861	48846257	55013819	104812053	64538526	44939362	56849778	45457247
Raw (Lane 2)	45447440	57362011	57875957	110669912	48737863	54564082	103793190	63803646	45268387	55930652	45420521
Raw (Lane 3)	46177093	57466765	57978466	110704549	48578805	54617398	105402722	64314228	45231788	56584564	45310625
Raw Total	137623098	172818379	173672981	333054322	146162925	164195299	314007965	192656400	135439537	169364994	136188393
Concatenated	137623098	172818379	173672981	NA	146162925	164195299	NA	192656400	135439537	169364994	136188393
CutAdapt	137623098	172818379	173672981	333054322	146162925	164195299	314007965	192656400	135439537	169364994	136188393
Trimmomatic	123570922	155399646	158099558	301999780	133061301	147385367	284519156	172659814	121788673	150528728	118981648
**Total # Reads and Base Pairs**											
Total reads	134877674	169672372	171132360	327718067	143704209	161534171	308796138	189078857	133135853	166025539	133880302
Total base pairs	38767289400	48760802700	49384787700	94457677050	41514826500	46337930700	88997294100	54260800650	38238678900	47483140050	37929292500
**Coverage**											
Pre-alignment	19.3836447	24.38040135	24.69239385	47.228838525	20.75741325	23.16896535	44.49864705	27.130400325	19.11933945	23.741570025	18.96464625
Post-alignment	17.6936	22.253	22.4177	42.9117	18.791	21.0385	40.2934	24.6062	16.7168	21.5514	17.0265
Rgadd	17.6936	22.253	22.4177	42.9117	18.791	21.0385	40.2934	24.6062	16.7168	21.5514	17.0265

MOLECULAR ECOLOGY

Dupsmarked	11.1401	14.5285	14.7101	28.4885	12.56583	13.1586	25.6738	15.8474	10.8892	13.118	10.0795
Mapped	11.1401	14.5285	14.7101	28.4885	12.56583	13.1586	25.6738	15.8474	10.8892	13.118	10.0795

MOLECULAR ECOLOGY

Supplementary Table 3: Sequencing statistics and quality for n = 14 Comal female samples at each stage of the data processing pipeline for raw reads as well as coverage throughout the alignment workflow. Coverage statistics are based on an alignment to *Pterygoplichthys* reference genome. Missing data represented with “NA”.

	F1	F2	F3	F4	F5	F6	F7	F8	F9	F10	F11	F12	F13	F14
**R1**														
Raw (Lane 1)	86668034	49512405	46050250	53739187	65457545	56513183	56214474	57153959	60084664	71213286	53654869	61030640	1659516	52790996
Raw (Lane 2)	86338560	49608572	46586636	53939799	65732532	56436564	56849145	57419200	60821368	71730634	54804995	60967326	1621271	52964777
Raw (Lane 3)	260256603	148651760	139111513	161591978	196771530	169445073	170132070	172396265	181663045	214545063	162762513	183518175	4913660	158440016
Raw Total	260256603	148651760	139111513	161591978	196771530	169445073	170132070	172396265	181663045	214545063	162762513	183518175	4913660	158440016
Concatenated	260256603	148651760	139111513	161591978	196771530	169186329	170132070	172396265	181663045	214545063	162762513	183518175	4913660	158440016
CutAdapt	260256603	148651760	139111513	161591978	196771530	169186329	170132070	172396265	181663045	214545063	162762513	183518175	4913660	158440016
Trimmomatic	236693353	132357216	121794353	142949766	179037953	152834646	153750874	153776679	162433890	194338869	145063828	165759537	3244158	142134689
**R2**														
Raw (Lane 1)	87250009	49530783	46474627	53912992	65581453	56495326	57068451	57823106	60757013	71601143	54302649	61520209	1632873	52684243
Raw (Lane 2)	86668034	49512405	46050250	53739187	65457545	56513183	56214474	57153959	60084664	71213286	53654869	61030640	1659516	52790996
Raw (Lane 3)	86338560	49608572	46586636	53939799	65732532	56436564	56849145	57419200	60821368	71730634	54804995	60967326	1621271	52964777
Raw Total	260256603	148651760	139111513	161591978	196771530	169445073	170132070	172396265	181663045	214545063	162762513	183518175	4913660	158440016
Concatenated	260256603	148651760	139111513	161591978	196771530	169186329	170132070	172396265	181663045	214545063	162762513	183518175	4913660	158440016
CutAdapt	260256603	148651760	139111513	161591978	196771530	169186329	170132070	172396265	181663045	214545063	162762513	183518175	4913660	158440016
Trimmomatic	236693353	132357216	121794353	142949766	179037953	152834646	153750874	153776679	162433890	194338869	145063828	165759537	3244158	142134689
**Total # Reads and Base Pairs**														
Total reads	255952469	146420904	136574553	159103132	194014611	166624021	166977498	169158353	178387039	210636906	159525669	180251770	4829240	156002500
Total base pairs	73896873300	41816718000	38755335900	45307934700	5595788560	47918800050	48109255800	48440254800	51123139350	60746366250	45688424550	51901696050	1211009700	44720578350
**Coverage**														
Pre-alignment	36.94843665	20.908359	19.37766795	22.653967	27.9289	23.9594	24.0546279	24.2201274	25.5615697	30.3731831	22.8442123	25.950848	0.60550485	22.3602892
Post-alignment	45.4356	25.8544	23.9325	27.7044	34.8116	29.6636	29.8765	29.9646	31.8259	37.7192	28.399	32.3052	0.420095	27.8452
Rgadd	45.4356	25.8544	23.9325	27.7044	34.8116	29.6636	29.8765	NA	NA	37.7192	28.399	32.3052	0.420095	27.8452

MOLECULAR ECOLOGY

Dupsmarked	30.1479	15.5564	13.7697	15.9037	21.4324	18.1494	18.7674	NA	NA	23.8033	17.4201	20.8629	0.136571	16.4025
Mapped	27.8366	15.5564	13.7697	15.9037	21.4324	18.1494	18.7674	NA	NA	23.8033	17.4201	20.8629	0.136571	16.4025

MOLECULAR ECOLOGY

Supplementary Table 4: Sequencing statistics and quality for n = 11 Comal male samples at each stage of the data processing pipeline for raw reads as well as coverage throughout the alignment workflow. Coverage statistics are based on an alignment to *Pterygoplichthys* reference genome. Missing data represented with “NA”.

	M1	M2	M3	M4	M5	M6	M7	M8	M9	M10	M11
**R1**											
Raw (Lane 1)	80400664	91124772	55300366	47676184	51318751	405392	61263559	50501246	86734914	49558232	59423985
Raw (Lane 2)	80457717	90558991	55643865	47562832	51206035	419393	61196837	50158405	86144998	49200962	58571925
Raw (Lane 3)	80497617	91778623	55573780	47627486	51249659	403952	61110197	50542523	87083793	49822342	59261057
Raw Total	241355998	273462386	166518011	142866502	153774445	1228737	183570593	151202174	259963705	148581536	177256967
Concatenated	241355998	273462386	166518011	142866502	153774445	1228737	183570593	151202174	259963705	148581536	177256967
CutAdapt	241355998	273462386	166518011	142866502	153774445	1228737	183570593	151202174	259963705	148581536	177256967
Trimmomatic	220029865	243910400	149379338	126889845	139313725	333109	166986267	134412036	232269895	131678541	157253050
**R2**											
Raw (Lane 1)	80400664	91124772	55300366	47676184	51318751	405392	61263559	50501246	86734914	49558232	59423985
Raw (Lane 2)	80457717	90558991	55643865	47562832	51206035	419393	61196837	50158405	86144998	49200962	58571925
Raw (Lane 3)	80497617	91778623	55573780	47627486	51249659	403952	61110197	50542523	87083793	49822342	59261057
Raw Total	241355998	273462386	166518011	142866502	153774445	1228737	183570593	151202174	259963705	148581536	177256967
Concatenated	241355998	273462386	166518011	142866502	153774445	1228737	183570593	151202174	259963705	148581536	177256967
CutAdapt Out	241355998	273462386	166518011	142866502	153774445	1228737	183570593	151202174	259963705	148581536	177256967
Trimmomatic	220029865	243910400	149379338	126889845	139313725	333109	166986267	134412036	232269895	131678541	157253050
**Total # Reads and Base Pairs**											
Total reads	237260386	268872119	164195611	140357385	151310027	1196868	180620970	148611051	255120714	145702190	173849105
Total base pairs	68593537650	76917377850	76917377850	40087084500	43593562800	229496550	52141085550	42453463050	73108591350	41607108650	49665323250
**Coverage**											
Pre-alignment	34.296768825	38.458688925	23.518121175	20.04354225	21.7967814	0.114748275	26.070542775	21.226731525	36.554295675	20.80354825	24.832661625
Post-alignment	42.3923	47.0671	29.2271	24.6898	27.17753	0.0125775	32.403	26.2098	45.164	25.7369	1.31785
Rgadd	42.3923	47.0671	29.2271	24.6898	27.17753	0.0125775	32.403	26.2098	45.164	25.7369	1.31785

Dupsmarked	27.0395	29.858	17.04	15.4519	16.8092	< 0.01	20.7421	15.7588	28.645	16.5556	1.06583
Mapped	27.0395	27.3564	17.04	15.4519	16.8092	< 0.01	20.7421	15.7588	28.1686	16.5556	1.06583

MOLECULAR ECOLOGY

Supplementary Table 5: Sequencing statistics and quality for n = 12 San Felipe female samples at each stage of the data processing pipeline for raw reads as well as coverage throughout the alignment workflow. Coverage statistics are based on an alignment to the *Hypostomus* reference genome. Missing data represented with “NA”.

	F1	F2	F3	F4	F5	F6	F7	F8	F9	F10	F11	F12
**R1**												
Raw (Lane 1)	67022775	53927235	59506051	59875048	50183131	72560731	65329521	52447709	65560660	54401051	70269964	47874293
Raw (Lane 2)	66387825	53720811	58716375	59472846	50745573	72096492	64389399	52258200	65346446	53905111	69493976	46924954
Raw (Lane 3)	66908235	54303957	58396518	59792781	50947829	72776282	64839116	52093393	65469764	54154987	69897752	47883628
Raw total	200318835	161952003	176618944	179140675	151876533	217433505	194558036	156799302	196376870	162461149	209661692	142682875
Concatenated	200318835	161952003	176618944	179140675	151876533	217433505	NA	156799302	196376870	162461149	209661692	142682875
CutAdapt Out (R1)	200318835	161952003	176618944	179140675	151876533	217433505	6205863	156799302	196376870	162461149	209661692	142682875
Trimmomatic Out (R1)	182481253	147088162	157310094	163065394	137070217	196864404	5500806	141565510	176998469	147807765	190523066	127098258
**R2**												
Raw (Lane 1)	67022775	53927235	59506051	59875048	50183131	72560731	65329521	52447709	65560660	54401051	70269964	47874293
Raw (Lane 2)	66387825	53720811	58716375	59472846	50745573	72096492	64389399	52258200	65346446	53905111	69493976	46924954
Raw (Lane 3)	66908235	54303957	58396518	59792781	50947829	72776282	64839116	52093393	65469764	54154987	69897752	47883628
Raw total	200318835	161952003	176618944	179140675	151876533	217433505	194558036	156799302	196376870	162461149	209661692	142682875
Concatenated	200318835	161952003	176618944	179140675	151876533	217433505	194558036	156799302	196376870	162461149	209661692	142682875
CutAdapt	200318835	161952003	176618944	179140675	151876533	217433505	194558036	156799302	196376870	162461149	209661692	142682875
Trimmomatic	182481253	147088162	157310094	163065394	137070217	196864404	5500806	141565510	176998469	147807765	190523066	127098258
**Total # Reads and Base Pairs**												
Total reads	197123433	159359980	173565960	176277793	149359792	213615210	6087700	154178790	192832411	159600663	206379953	139704903
Total base pairs	56940702900	45967221300	49631408100	50901478050	42964501350	61571942100	1738275900	44361645000	55474632000	46111264200	59535452850	40020474150
**Coverage**												
Pre-alignment	28.47035145	22.98361065	24.81570405	25.450739025	21.482250675	30.78597105	0.86913795	22.1808225	27.737316	23.0556321	29.767726425	20.010237075
Post-alignment	25.9692	20.9314	22.3956	23.1399	19.4778	1.00695	0.785636	20.1356	25.1563	20.8327	27.0586	18.1433

Rgadd	25.9692	20.9314	22.3956	23.1399	19.4778	1.00695	0.785636	20.1356	25.1563	20.8327	27.0586	18.1433
Dupsmarked	16.4975	13.1827	14.9703	14.8895	12.841	0.85556	0.575402	13.685	15.8028	13.8601	17.1272	11.6785
Mapped	16.4975	13.1827	14.9703	14.8895	12.841	0.85556	0.575402	13.685	15.8028	13.8601	17.1272	11.6785

MOLECULAR ECOLOGY

Supplementary Table 6: Sequencing statistics and quality for n = 12 San Felipe male samples at each stage of the data processing pipeline for raw reads as well as coverage throughout the alignment workflow. Coverage statistics are based on an alignment to the *Hypostomus* reference genome.

	M1	M2	M3	M4	M5	M6	M7	M8	M9	M10	M11	M12
**R1**												
Raw (Lane 1)	48005111	48075861	47334750	56989231	57886779	60957699	50340051	58815795	49826078	62228842	45018836	69585864
Raw (Lane 2)	47673790	47726159	47083943	56453061	57085890	60423899	49713203	58127996	49159683	61936925	44837685	69255325
Raw (Lane 3)	47899706	47965369	47300140	57729324	58210880	61302924	50394569	58576563	49760100	62379896	45147996	69583319
Raw total	143578607	143767389	141718833	171171616	173183549	182684522	150447823	175520354	148745861	186545663	135004517	208424508
Concatenated	143578607	143767389	141718833	171171616	173183549	182684522	150447823	175520354	148745861	186545663	135004517	208424508
CutAdapt	143578607	143767389	141718833	171171616	173183549	182684522	150447823	175520354	148745861	186545663	135004517	208424508
Trimmomatic	129256444	128834416	127863223	154927860	151316476	164284172	135558233	157982891	133069559	171010601	121988514	191560284
**R2**												
Raw (Lane 1)	48005111	48075861	47334750	56989231	57886779	60957699	50340051	58815795	49826078	62228842	45018836	69585864
Raw (Lane 2)	47673790	47726159	47083943	56453061	57085890	60423899	49713203	58127996	49159683	61936925	44837685	69255325
Raw (Lane 3)	47899706	47965369	47300140	57729324	58210880	61302924	50394569	58576563	49760100	62379896	45147996	69583319
Raw total	143578607	143767389	141718833	171171616	173183549	182684522	150447823	175520354	148745861	186545663	135004517	208424508
Concatenated	143578607	143767389	141718833	171171616	173183549	182684522	150447823	175520354	148745861	186545663	135004517	208424508
CutAdapt	143578607	143767389	141718833	171171616	173183549	182684522	150447823	175520354	148745861	186545663	135004517	208424508
Trimmomatic	129256444	128834416	127863223	154927860	151316476	164284172	135558233	157982891	133069559	171010601	121988514	191560284
**Total # Reads and Base Pairs**												
Total reads	132554894	141021005	139008767	168236913	170181103	179366920	147894626	172189810	145971136	183821054	132554894	205198911
Total base pairs	38181511200	49887193350	40030798500	48474715950	48224636850	51547663800	42517928850	49525905150	41856104250	53224748250	38181511200	59513879250
**Coverage**												
Pre-alignment	19.0907556	24.943596675	20.01539925	24.237357975	24.11231843	25.7738319	21.25896443	24.762952558	20.92805213	26.61237413	19.0907556	29.75693963
Post-alignment	18.3047	18.2969	18.111	22.1114	21.6624	0.846384	19.4021	22.4755	19.0625	24.2161	17.3133	26.8891
Rgadd	18.3047	18.2969	18.111	22.1114	21.6624	0.846384	19.4021	22.4755	19.0625	24.2161	17.3133	26.8891
Dupsmarked	12.099	11.9963	11.6464	13.5107	13.5242	0.72086	12.0658	14.7351	11.961	15.6174	11.5379	18.0543

MOLECULAR ECOLOGY

Mapped	12.099	11.9963	11.6464	13.5107	13.5242	0.72086	12.0658	14.7351	11.961	15.6174	11.5379	18.0543

MOLECULAR ECOLOGY

Supplementary Table 7: Number of repeat sequences present in each repeat family in *Hypostomus* sp. reference genome as identified by RepeatModeler and RepeatMasker.

	Number of repeats in assembly
**DNA**	33
**DNA/Academ-1**	1
**DNA/CMC-Chapaev-3**	7
**DNA/CMC-EnSpm**	14
**DNA/Crypton**	4
**DNA/Crypton-A**	4
**DNA/Crypton-V**	1
**DNA/Ginger-1**	3
**DNA/hAT**	4
**DNA/hAT-Ac**	128
**DNA/hAT-Blackjack**	8
**DNA/hAT-Charlie**	37
**DNA/hAT-hAT5**	5
**DNA/hAT-hATm**	2
**DNA/hAT-Tip100**	19
**DNA/IS3EU**	5
**DNA/Kolobok-T2**	1
**DNA/Maverick**	3
**DNA/MULE-MuDR**	1
**DNA/P**	2
**DNA/PIF**	1
**DNA/PIF-Harbinger**	40
**DNA/PIF-ISL2EU**	4

**DNA/PiggyBac**	11
**DNA/Sola-1**	1
**DNA/Sola-2**	1
**DNA/TcMar**	2
**DNA/TcMar-Fot1**	3
**DNA/TcMar-ISRm11**	6
**DNA/TcMar-Mariner**	1
**DNA/TcMar-Tc1**	187
**DNA/TcMar-Tc2**	2
**DNA/TcMar-Tigger**	75
**LINE/I**	25
**LINE/L1**	20
**LINE/L1-Tx1**	8
**LINE/L2**	68
**LINE/Penelope**	13
**LINE/R2-Hero**	6
**LINE/R2-NeSL**	1
**LINE/Rex-Babar**	50
**LINE/RTE-BovB**	18
**LINE/RTE-X**	11
**LTR**	5
**LTR/Copia**	1
**LTR/ERV1**	22
**LTR/ERVK**	1
**LTR/Gypsy**	46

**LTR/Ngaro**	17
**LTR/Pao**	6
**RC/Helitron**	7
**Retroposon**	1
**rRNA**	2
**Satellite**	6
**Simple_repeat**	24
**SINE?**	2
**SINE/5S**	1
**SINE/B2**	2
**SINE/ID**	5
**SINE/MIR**	6
**SINE/tRNA**	4
**SINE/tRNA-RTE**	5
**SINE/tRNA-V-RTE**	1
**tRNA**	13
**Unknown**	1295

MOLECULAR ECOLOGY

Supplementary Table 8: List of cytochrome oxidase I (COI) gene sequences from Genbank used for mitochondrial phylogeny.

Accession No.	Taxon
AP012021.1	*Pterygoplichthys disjunctivus*
EU359422.1	*Hypostomus boulengeri*
FJ434521.1	*Hypostomus ancistroides*
GQ225397.1	*Hypostomus cochliodon*
GU701547.1	*Pterygoplichthys ambrosettii*
GU701550.1	*Pterygoplichthys ambrosettii*
GU701551.1	*Pterygoplichthys ambrosettii*
GU701680.1	*Hypostomus regani*
GU701683.1	*Hypostomus paulinus*
GU701684.1	*Hypostomus paulinus*
GU701687.1	*Hypostomus paulinus*
GU701688.1	*Hypostomus nigromaculatus*
GU701689.1	*Hypostomus nigromaculatus*
GU701691.1	*Hypostomus* sp.
GU701692.1	*Hypostomus myersi*
GU701693.1	*Hypostomus myersi*
GU701694.1	*Hypostomus microstomus*
GU701695.1	*Hypostomus regani*
GU701696.1	*Hypostomus myersi*
GU701697.1	*Hypostomus* sp.
GU701698.1	*Hypostomus microstomus*
GU701699.1	*Hypostomus iheringii*
GU701700.1	*Hypostomus topavae*
GU701705.1	*Hypostomus heraldoi*
GU701706.1	*Hypostomus hermanni*

Accession No.	Taxon
GU701707.1	*Hypostomus hermanni*
GU701708.1	*Hypostomus heraldoi*
GU701709.1	*Hypostomus heraldoi*
GU701710.1	*Hypostomus heraldoi*
GU701711.1	*Hypostomus heraldoi*
GU701713.1	*Hypostomus derbyi*
GU701715.1	*Hypostomus brevis*
GU701717.1	*Hypostomus commersoni*
GU701719.1	*Hypostomus commersoni*
GU701720.1	*Hypostomus commersoni*
GU701721.1	*Hypostomus brevis*
GU701722.1	*Hypostomus commersoni*
GU701724.1	*Hypostomus brevis*
GU701725.1	*Hypostomus ancistroides*
GU701726.1	*Hypostomus ancistroides*
GU701727.1	*Hypostomus ancistroides*
GU701728.1	*Hypostomus albopunctatus*
GU701729.1	*Hypostomus ancistroides*
GU701730.1	*Hypostomus albopunctatus*
GU701731.1	*Hypostomus albopunctatus*
GU701732.1	*Hypostomus regani*
GU701877.1	*Hypostomus strigaticeps*
GU701953.1	*Hypostomus strigaticeps*
GU701954.1	*Hypostomus strigaticeps*
GU701963.1	*Hypostomus regani*

MOLECULAR ECOLOGY

Accession No.	Taxon
GU701966.1	*Hypostomus regani*
GU702244.1	*Hypostomus affinis*
GU702253.1	*Hypostomus affinis*
GU702254.1	*Hypostomus affinis*
GU702261.1	*Hypostomus auroguttatus*
GU702262.1	*Hypostomus auroguttatus*
HM376401.1	*Hypostomus cochliodon*
HM405132.1	*Hypostomus alatus*
HM405133.1	*Hypostomus alatus*
HM405134.1	*Hypostomus alatus*
HM405209.1	*Pterygoplichthys etentaculatus*
HM405210.1	*Pterygoplichthys etentaculatus*
HQ573337.1	*Pterygoplichthys disjunctivus*
HQ573338.1	*Pterygoplichthys sp.*
HQ600829.1	*Hypostomus alatus*
HQ682719.1	*Pterygoplichthys pardalis*
HQ682720.1	*Pterygoplichthys pardalis*
JF498719.1	*Pterygoplichthys disjunctivus*
JF498720.1	*Pterygoplichthys disjunctivus*
JF498721.1	*Pterygoplichthys disjunctivus*
JF498722.1	*Pterygoplichthys disjunctivus*
JF498723.1	*Pterygoplichthys disjunctivus*
JF498724.1	*Pterygoplichthys disjunctivus*
JF498754.1	*Pterygoplichthys pardalis*
JF769356.1	*Pterygoplichthys disjunctivus*

Accession No.	Taxon
JF769357.1	*Pterygoplichthys pardalis*
JF769358.1	*Pterygoplichthys pardalis*
JF769359.1	*Pterygoplichthys pardalis*
JF769360.1	*Pterygoplichthys pardalis*
JF769361.1	*Pterygoplichthys pardalis*
JF769362.1	*Pterygoplichthys pardalis*
JN026849.1	*Hypostomus plecostomus*
JN026850.1	*Hypostomus plecostomus*
JN026851.1	*Hypostomus plecostomus*
JN028316.1	*Pterygoplichthys* cf. *disjunctivus*
JN988933.1	*Hypostomus brevis*
JN988934.1	*Hypostomus brevis*
JN988935.1	*Hypostomus brevis*
JN988937.1	*Hypostomus commersoni*
JN988938.1	*Hypostomus commersoni*
JN988939.1	*Hypostomus derbyi*
JN988940.1	*Hypostomus derbyi*
JN988943.1	*Hypostomus iheringii*
JN988944.1	*Hypostomus iheringii*
JN988945.1	*Hypostomus margaritifer*
JN988947.1	*Hypostomus margaritifer*
JN988950.1	*Hypostomus regani*
JN988952.1	*Hypostomus topavae*
JQ667566.1	*Pterygoplichthys pardalis*
JQ667567.1	*Pterygoplichthys pardalis*

Accession No.	Taxon
JQ667568.1	*Pterygoplichthys pardalis*
JX111769.1	*Hypostomus commersoni*
JX111770.1	*Hypostomus commersoni*
JX111771.1	*Hypostomus commersoni*
JX443490.1	*Hypostomus ancistroides*
JX443491.1	*Hypostomus ancistroides*
JX443494.1	*Hypostomus strigaticeps*
JX443495.1	*Hypostomus strigaticeps*
JX443496.1	*Hypostomus strigaticeps*
JX443497.1	*Hypostomus nigromaculatus*
JX443498.1	*Hypostomus nigromaculatus*
JX443499.1	*Hypostomus nigromaculatus*
JX443500.1	*Hypostomus nigromaculatus*
JX495614.1	*Pterygoplichthys* cf. *pardalis*
JX495615.1	*Pterygoplichthys* cf. *pardalis*
KC170030.1	*Pterygoplichthys disjunctivus*
KC170031.1	*Pterygoplichthys disjunctivus*
KC170032.1	*Pterygoplichthys disjunctivus*
KC170033.1	*Pterygoplichthys disjunctivus*
KC170034.1	*Pterygoplichthys disjunctivus*
KC170035.1	*Pterygoplichthys disjunctivus*
KF410695.1	*Hypostomus plecostomus*
KJ720692.1	*Hypostomus plecostomus*
KJ720697.1	*Hypostomus punctatus*
KM104508.1	*Hypostomus microstomus*

MOLECULAR ECOLOGY

Accession No.	Taxon
KM104509.1	*Hypostomus ancistroides*
KM576100.1	*Hypostomus plecostomus*
KM897223.1	*Hypostomus regani*
KM897247.1	*Hypostomus regani*
KM897277.1	*Hypostomus ancistroides*
KM897343.1	*Hypostomus strigaticeps*
KM897631.1	*Hypostomus regani*
KP772577.1	*Hypostomus carinatus*
KP772585.1	*Hypostomus macushi*
KR491517.1	*Pterygoplichthys multiradiatus*
KT239003.1	*Pterygoplichthys anisitsi*
KT239004.1	*Pterygoplichthys anisitsi*
KT239005.1	*Pterygoplichthys anisitsi*
KT239012.1	*Hypostomus* aff. *plecostomus*
KT239013.1	*Hypostomus affinis*
KT239016.1	*Pterygoplichthys pardalis*
KT952445.1	*Hypostomus* sp.
KU288820.1	*Pterygoplichthys anisitsi*
KU568996.1	*Pterygoplichthys* sp.
KU568997.1	*Pterygoplichthys gibbiceps*
KU568997.1	*Pterygoplichthys gibbiceps*
KX087171.1	*Hypostomus* sp.
KX087181.1	*Pterygoplichthys* sp.
KY349029.1	*Hypostomus jaguribensis*
KY349030.1	*Hypostomus jaguribensis*

MOLECULAR ECOLOGY

Accession No.	Taxon
KY349037.1	*Hypostomus pusarum*
KY349038.1	*Hypostomus pusarum*
KY349039.1	*Hypostomus* sp.
KY349040.1	*Hypostomus* sp.
KY349041.1	*Hypostomus* sp.
KY349042.1	*Hypostomus* sp.
MG825006.1	*Hypostomus affinis*
MG825026.1	*Hypostomus luetkeni*
MH087048.1	*Pterygoplichthys anisitsi*
MK026008.1	*Hypostomus francisci*
MK049441.1	*Pterygoplichthys anisitsi*
MK355286.1	*Hypostomus* sp.
MK464029.1	*Hypostomus* sp.
MK464032.1	*Hypostomus* sp.
MK464054.1	*Hypostomus* sp.
MK464060.1	*Hypostomus* sp.
MK464075.1	*Hypostomus* sp.
MK464108.1	*Hypostomus* sp.
MK464112.1	*Hypostomus* sp.
MK464113.1	*Hypostomus* sp.
MK464114.1	*Hypostomus* sp.
MK464115.1	*Hypostomus* sp.
MK464116.1	*Hypostomus* sp.
MK464117.1	*Hypostomus* sp.
MK464118.1	*Hypostomus* sp.

Accession No.	Taxon
MK464119.1	*Hypostomus* sp.
MK464120.1	*Hypostomus* sp.
MK464141.1	*Hypostomus* sp.
MK464142.1	*Hypostomus* sp.
MK464143.1	*Hypostomus* sp.
MK464144.1	*Hypostomus* sp.
MK464145.1	*Hypostomus* sp.
MK464146.1	*Hypostomus* sp.
MK464147.1	*Hypostomus* sp.
MK464163.1	*Hypostomus* sp.
MK464172.1	*Hypostomus* sp.
MK572527.1	*Pterygoplichthys* sp.
MK628372.1	*Pterygoplichthys anisitsi*
MK660623.1	*Hypostomus variipictus*
MK660624.1	*Hypostomus variipictus*
MK959837.1	*Pterygoplichthys scrophus*
MK959838.1	*Hypostomus microstomus*
MK959839.1	*Hypostomus cochliodon*
MK959840.1	*Hypostomus arecuta*
MK959841.1	*Hypostomus luteomaculatus*
MK959843.1	*Hypostomus regani*
MK959844.1	*Hypostomus paranensis*
MK959845.1	*Hypostomus formosae*
MK959846.1	*Hypostomus boulengeri*
MK959847.1	*Hypostomus* sp.

MOLECULAR ECOLOGY

Accession No.	Taxon
MK959848.1	*Hypostomus borellii*
MK959849.1	*Hypostomus ericae*
MK959850.1	*Hypostomus* sp.
MK959851.1	*Hypostomus interruptus*
MK959852.1	*Hypostomus* sp.
MK959853.1	*Hypostomus affinis*
MK959854.1	*Hypostomus asperatus*
MK959855.1	*Hypostomus* sp.
MK959856.1	*Hypostomus* sp.
MK959858.1	*Hypostomus* sp.
MK959859.1	*Hypostomus* sp.
MK959860.1	*Hypostomus aspilogaster*
MK959861.1	*Hypostomus* sp.
MK959862.1	*Hypostomus watwata*
MK959863.1	*Hypostomus taphorni*
MK959864.1	*Hypostomus hemiurus*
MK959865.1	*Hypostomus albopunctatus*
MK959866.1	*Hypostomus isbrueckeri*
MK959867.1	*Hypostomus* sp.
MK959868.1	*Hypostomus fonchii*
MK959869.1	*Hypostomus* sp.
MK959870.1	*Hypostomus* sp.
MK959871.1	*Hypostomus oculeus*
MK959872.1	*Hypostomus* sp.
MK959873.1	*Hypostomus latifrons*

Accession No.	Taxon
MK959874.1	*Hypostomus plecostomus*
MK959875.1	*Hypostomus nigromaculatus*
MK959876.1	*Hypostomus luteus*
MK959878.1	*Pterygoplichthys multiradiatus*
MK959879.1	*Aphanotorulus ammophilus*
MK959880.1	*Pterygoplichthys zuliaensis*
MK959881.1	*Hypostomus laplatae*
MK959882.1	*Hypostomus spiniger*
MK959884.1	*Hypostomus myersi*
MK959885.1	*Hypostomus derbyi*
MK959886.1	*Hypostomus uruguayensis*
MK959887.1	*Hypostomus ternetzi*
MK959888.1	*Hypostomus hondae*
MK959889.1	*Hypostomus plecostomoides*
MK959890.1	*Hypostomus ancistroides*
MK959891.1	*Hypostomus mutucae*
MK959892.1	*Hypostomus* sp.
MK959893.1	*Hypostomus ancistroides*
MN854452.1	*Hypostomus* sp.
MN854453.1	*Hypostomus scabriceps*
MN854454.1	*Hypostomus* cf. *borellii*
MN854455.1	*Hypostomus taphorni*
MN854456.1	*Hypostomus boulengeri*
MN854457.1	*Hypostomus hemiurus*
MN854458.1	*Hypostomus* sp.

MOLECULAR ECOLOGY

Accession No.	Taxon
MN854459.1	*Hypostomus micromaculatus*
MN854460.1	*Hypostomus* sp.
MN854461.1	*Hypostomus* sp.
MN854462.1	*Hypostomus paranensis*
MN854463.1	*Hypostomus basilisko*
MN854464.1	*Hypostomus* cf. *borellii*
MN854465.1	*Hypostomus* sp.
MN854466.1	*Hypostomus* sp.
MN854467.1	*Hypostomus taphorni*
MN854468.1	*Hypostomus commersoni*
MN854469.1	*Hypostomus commersoni*
MN854470.1	*Hypostomus* sp.
MN854471.1	*Hypostomus* sp.
MN854472.1	*Hypostomus* sp.
MN854473.1	*Hypostomus crassicauda*
MN854474.1	*Hypostomus* sp.
MN854475.1	*Hypostomus* sp.
MN854476.1	*Hypostomus micromaculatus*
MN854477.1	*Hypostomus gymnorhynchus*
MN854478.1	*Hypostomus gymnorhynchus*
MN854479.1	*Hypostomus gymnorhynchus*
MN854480.1	*Hypostomus gymnorhynchus*
MN854481.1	*Hypostomus gymnorhynchus*
MN854482.1	*Hypostomus* sp.
MN854483.1	*Hypostomus corantijni*

MOLECULAR ECOLOGY

Accession No.	Taxon
MN854484.1	*Hypostomus plecostomus*
MN854485.1	*Hypostomus* sp.
MN854486.1	*Hypostomus plecostomus*
MN854487.1	*Hypostomus* sp.
MN854488.1	*Hypostomus corantijni*
MN854489.1	*Hypostomus plecostomus*
MN854490.1	*Hypostomus watwata*
MN854491.1	*Hypostomus niceforoi*
MN854492.1	*Hypostomus oculeus*
MN854493.1	*Hypostomus nematopterus*
MN854494.1	*Hypostomus* sp.
MN854495.1	*Hypostomus mutucae*
MN854496.1	*Hypostomus uruguayensis*
MN854497.1	*Hypostomus faveolus*
MN854498.1	*Hypostomus uruguayensis*
MN854499.1	*Hypostomus faveolus*
MN854500.1	*Hypostomus pusarum*
MN854501.1	*Aphanotorulus unicolor*
MN854502.1	*Aphanotorulus unicolor*
MN854503.1	*Hypostomus aspilogaster*
MN854504.1	*Aphanotorulus emarginatus*
MN854505.1	*Aphanotorulus emarginatus*
MN854506.1	*Aphanotorulus emarginatus*
MN854507.1	*Aphanotorulus emarginatus*
MN854508.1	*Hypostomus hoplonites*

Accession No.	Taxon
MN854509.1	*Hypostomus* sp.
MN854510.1	*Hypostomus gymnorhynchus*
MN854511.1	*Hypostomus* sp.
MN854512.1	*Hypostomus basilisko*
MN854513.1	*Hypostomus holostictus*
MN854514.1	*Hypostomus holostictus*
MN854515.1	*Hypostomus robinii*
MN854516.1	*Hypostomus hemicochliodon*
MN854517.1	*Hypostomus plecostomoides*
MN854518.1	*Hypostomus sculpodon*
MN854519.1	*Hypostomus sculpodon*
MN854520.1	*Hypostomus soniae*
MN854521.1	*Hypostomus paucimaculatus*
MN854522.1	*Aphanotorulus* sp.
MN854523.1	*Hypostomus pyrineusi*
MN854524.1	*Hypostomus* sp.
MN854525.1	*Hypostomus* sp.
MN854526.1	*Hypostomus francisci*
MN854527.1	*Aphanotorulus emarginatus*
MN854528.1	*Aphanotorulus emarginatus*
MN854529.1	*Pseudacanthicus* cf. *leopardus*
MN854530.1	*Pterygoplichthys joselimaianus*
MN854531.1	*Hypostomus* aff. *regani*
MN854532.1	*Hypostomus plecostomus*
MN854533.1	*Hypostomus plecostomus*

MOLECULAR ECOLOGY

Accession No.	Taxon
MN854534.1	*Hypancistrus debilittera*
MN854535.1	*Baryancistrus demantoides*
MN854536.1	*Hypostomus* sp.
MN854537.1	*Peckoltia* sp.
MN854538.1	*Panaque nigrolineatus*
MN854539.1	*Hypostomus gymnorhynchus*
MN854540.1	*Hypostomus gymnorhynchus*
MN854541.1	*Panaqolus albivermis*
MN854542.1	*Panaqolus* sp.
MN854543.1	*Parancistrus nudiventris*
MN854544.1	*Aphanotorulus* sp.
MN854545.1	*Hypostomus topavae*
MN854546.1	*Pterygoplichthys* aff. *ambrosettii*
MN854547.1	*Hypostomus* sp.
MN854548.1	*Hypostomus* sp.
MN854549.1	*Hypostomus meleagris*
MN854550.1	*Hypostomus oculeus*
MN854551.1	*Pterygoplichthys* aff. *ambrosettii*
MN854552.1	*Pterygoplichthys* aff. *ambrosettii*
MN854553.1	*Hypostomus* sp.
MN854554.1	*Aphanotorulus emarginatus*
MN854555.1	*Pterygoplichthys scrophus*
MN854556.1	*Pterygoplichthys pardalis*
MN854557.1	*Isorineloricaria spinosissima*
MN854558.1	*Peckoltia braueri*

Accession No.	Taxon
MN854559.1	*Peckoltia sabaji*
MN854560.1	*Hypostomus auroguttatus*
MN854561.1	*Hypostomus scabriceps*
MN854562.1	*Pterygoplichthys gibbiceps*
MN854563.1	*Hypostomus coppenamensis*
MN854564.1	*Hypostomus coppenamensis*
MN854565.1	*Hypostomus watwata*
MN854566.1	*Peckoltia cavatica*
MN854567.1	*Peckoltia vittata*
MN854568.1	*Panaque bathyphilus*
MN854569.1	*Hypostomus* sp.
MN854570.1	*Pterygoplichthys pardalis*
MN854571.1	*Hypostomus arecuta*
MN854572.1	*Pterygoplichthys lituratus*
MN854573.1	*Pterygoplichthys ambrosettii*
MN854574.1	*Hypostomus saramaccensis*
MN854575.1	*Peckoltia oligospila*
MN854576.1	*Hypostomus scabriceps*
MN854577.1	*Hypostomus watwata*
MN854578.1	*Scobinancistrus* cf. *pariolispos*
MN854579.1	*Hypostomus* aff. *weberi*
MN854580.1	*Hypostomus macushi*
MN854581.1	*Hypostomus macushi*
MN854582.1	*Hypostomus macushi*
MN854583.1	*Hypostomus hoplonites*

Accession No.	Taxon
MN854584.1	*Hypostomus crassicauda*
MN854585.1	*Aphanotorulus emarginatus*
MN854586.1	*Hypostomus coppenamensis*
MN854587.1	*Hypostomus* sp.
MN854588.1	*Hypostomus saramaccensis*
MN854589.1	*Hypostomus* sp.
MN854590.1	*Hypostomus* sp.
MN854591.1	*Hypostomus* sp.
MN854592.1	*Hypostomus strigaticeps*
MN854593.1	*Hypostomus pusarum*
MN854594.1	*Hypostomus* sp.
MN854595.1	*Hypostomus* sp.
MN854596.1	*Hypostomus carinatus*
MN854597.1	*Hypostomus* sp.
MN854598.1	*Hypostomus* sp.
MN854599.1	*Hypostomus hoplonites*
MN854600.1	*Hypostomus* sp.
MN854601.1	*Hypostomus* sp.
MN854602.1	*Hypostomus* sp.
MN854603.1	*Hypostomus* sp.
MN854604.1	*Hypostomus* sp.
MN854605.1	*Hypostomus paulinus*
MN854606.1	*Hypostomus* sp.
MN854607.1	*Hypostomus carinatus*
MN854608.1	*Hypostomus weberi*

Accession No.	Taxon
MN854609.1	*Hypostomus* aff. *pyrineusi*
MN854610.1	*Hypostomus weberi*
MN854611.1	*Hypostomus carinatus*
MN854612.1	*Hypostomus goyazensis*
MN854613.1	*Hypostomus ericae*
MN854614.1	*Hypostomus* sp.
MN854615.1	*Hypostomus paucipunctatus*
MN854616.1	*Hypostomus* sp.
MN854617.1	*Hypostomus* sp.
MN854618.1	*Hypostomus iheringii*
MN854619.1	*Hypostomus kopeyaka*
MN854620.1	*Hypostomus carinatus*
MN854621.1	*Hypostomus plecostomoides*
MN854622.1	*Hypostomus* sp.
MN854623.1	*Hypostomus weberi*
MN854624.1	*Hypostomus rhantos*
MN854625.1	*Hypostomus spiniger*
MN854626.1	*Hypostomus* sp.
MN854627.1	*Hypostomus* sp.
MN854628.1	*Ancistomus snethlageae*
MN854629.1	*Hypostomus* sp.
MN854630.1	*Hypostomus plecostomus*
MN854631.1	*Hypostomus* aff. *piratatu*
MN854632.1	*Hypostomus* sp.
MN854633.1	*Hypostomus albopunctatus*

MOLECULAR ECOLOGY

Accession No.	Taxon
MN880037.1	*Hypostomus ancistroides*
MN880038.1	*Hypostomus ancistroides*
MN880039.1	*Hypostomus ancistroides*
MN880040.1	*Hypostomus ancistroides*
MN880041.1	*Hypostomus ancistroides*
MN880042.1	*Hypostomus iheringii*
MN880043.1	*Hypostomus iheringii*
MN880044.1	*Hypostomus albopunctatus*
MN880045.1	*Hypostomus albopunctatus*
MN880046.1	*Hypostomus albopunctatus*
MN880047.1	*Hypostomus albopunctatus*
MN880048.1	*Hypostomus albopunctatus*
MT066232.1	*Hypostomus ancistroides*
MT081402.1	*Hypostomus* sp.
MT396945.1	*Hypostomus* sp.
MT666008.1	*Hypostomus watwata*
MT884726.1	*Pterygoplichthys* sp.
MW379881.1	*Hypostomus leucophaeus*
MW379882.1	*Hypostomus jaguar*
MW379883.1	*Hypostomus jaguar*
MW379884.1	*Hypostomus leucophaeus*
MW379885.1	*Hypostomus brevicauda*
MW379886.1	*Hypostomus brevicauda*
MW379887.1	*Hypostomus leucophaeus*
MW379888.1	*Hypostomus leucophaeus*

MOLECULAR ECOLOGY

Accession No.	Taxon
MW379889.1	*Hypostomus leucophaeus*
MW379890.1	*Hypostomus jaguar*
MW379891.1	*Hypostomus jaguar*
MW379892.1	*Hypostomus wuchereri*
MW379893.1	*Hypostomus* aff. *unae*
MW379894.1	*Hypostomus* aff. *unae*
MW379895.1	*Hypostomus* aff. *unae*
MW379896.1	*Hypostomus* sp.
MW379897.1	*Hypostomus* sp.
MW379898.1	*Hypostomus* aff. *unae*
MW379899.1	*Hypostomus* aff. *unae*
MW379900.1	*Hypostomus* aff. *unae*
MW379901.1	*Hypostomus* sp.
MW379902.1	*Hypostomus* sp.
MW379903.1	*Pterygoplichthys chrysostiktos*
MW379904.1	*Pterygoplichthys chrysostiktos*
MW379905.1	*Hypostomus jaguar*
MW379906.1	*Hypostomus jaguar*
MW379907.1	*Hypostomus jaguar*
MW379908.1	*Hypostomus jaguar*
MW379909.1	*Pterygoplichthys chrysostiktos*
MW379910.1	*Hypostomus wuchereri*
MW379911.1	*Hypostomus brevicauda*
MW379912.1	*Hypostomus wuchereri*
MW379913.1	*Hypostomus wuchereri*

Accession No.	Taxon
MW379914.1	*Hypostomus wuchereri*
MW379915.1	*Hypostomus wuchereri*
MW379916.1	*Hypostomus brevicauda*
MW379917.1	*Hypostomus brevicauda*
MW379918.1	*Hypostomus leucophaeus*
MW379919.1	*Hypostomus wuchereri*
MW379920.1	*Hypostomus wuchereri*
MW379921.1	*Hypostomus wuchereri*
MW379922.1	*Hypostomus leucophaeus*
MW379923.1	*Hypostomus wuchereri*
MW379924.1	*Hypostomus wuchereri*
MW379925.1	*Hypostomus wuchereri*
MW379926.1	*Hypostomus brevicauda*
MW379927.1	*Hypostomus brevicauda*
MW379928.1	*Hypostomus wuchereri*
MW379929.1	*Hypostomus wuchereri*
MW379930.1	*Hypostomus wuchereri*
MW379931.1	*Hypostomus* sp.
MW379932.1	*Hypostomus* sp.
MW379933.1	*Hypostomus nigrolineatus*
MW379934.1	*Hypostomus nigrolineatus*
MW379935.1	*Hypostomus nigrolineatus*
MW379936.1	*Hypostomus nigrolineatus*
MW379937.1	*Hypostomus wuchereri*
MW379938.1	*Hypostomus wuchereri*

Accession No.	Taxon
MW379939.1	*Hypostomus* aff. *unae*
MW379940.1	*Hypostomus* aff. *unae*
MW379941.1	*Hypostomus affinis*
MW379942.1	*Pterygoplichthys* aff. *ambrosettii*
MW379943.1	*Hypostomus wuchereri*
MW379944.1	*Hypostomus wuchereri*
MW379945.1	*Hypostomus wuchereri*
MW379946.1	*Hypostomus wuchereri*
MW379947.1	*Hypostomus wuchereri*
MW379948.1	*Hypostomus wuchereri*
MW379949.1	*Hypostomus wuchereri*
MW379950.1	*Hypostomus francisci*
MW379951.1	*Hypostomus francisci*
MW379952.1	*Hypostomus francisci*
MW379953.1	*Hypostomus francisci*
MW379954.1	*Hypostomus francisci*
MW379955.1	*Hypostomus francisci*
MW379956.1	*Hypostomus francisci*
MW379957.1	*Hypostomus* cf. *macrops*
MW379958.1	*Hypostomus francisci*
MW379959.1	*Hypostomus francisci*
MW379960.1	*Hypostomus francisci*
MW379961.1	*Hypostomus francisci*
MW379962.1	*Hypostomus velhochico*
MW379963.1	*Hypostomus velhochico*

Accession No.	Taxon
MW379964.1	*Hypostomus francisci*
MW379965.1	*Hypostomus francisci*
MW379966.1	*Hypostomus francisci*
MW379967.1	*Hypostomus francisci*
MW379968.1	*Hypostomus velhochico*
MW379969.1	*Hypostomus* cf. *macrops*
MW379970.1	*Hypostomus* cf. *macrops*
MW379971.1	*Hypostomus* cf. *paulinus*
MW379972.1	*Hypostomus margaritifer*
MW379973.1	*Hypostomus* cf. *macrops*
MW379974.1	*Hypostomus* cf. *macrops*
MW379975.1	*Hypostomus* sp.
MW379976.1	*Hypostomus* sp.
MW379977.1	*Hypostomus* sp.
MW379978.1	*Hypostomus* sp.
MW379979.1	*Hypostomus* sp.
MW379980.1	*Hypostomus* sp.
MW379981.1	*Hypostomus* sp.
MW379982.1	*Hypostomus* aff. *affinis*
MW379983.1	*Hypostomus* aff. *affinis*
MW379984.1	*Hypostomus* aff. *affinis*
MW379985.1	*Hypostomus francisci*
MW379986.1	*Hypostomus francisci*
MW379987.1	*Hypostomus luetkeni*
MW379988.1	*Hypostomus affinis*

MOLECULAR ECOLOGY

Accession No.	Taxon
MW379989.1	*Hypostomus scabriceps*
MW379990.1	*Hypostomus luetkeni*
MW379991.1	*Hypostomus scabriceps*
MW379992.1	*Hypostomus scabriceps*
MW379993.1	*Hypostomus scabriceps*
MW379994.1	*Hypostomus affinis*
MW379995.1	*Hypostomus affinis*
MW379996.1	*Hypostomus affinis*
MW379997.1	*Hypostomus affinis*
MW379998.1	*Hypostomus* sp.
MW379999.1	*Hypostomus francisci*
MW380000.1	*Hypostomus alatus*
MW380001.1	*Hypostomus luetkeni*
MW380002.1	*Hypostomus affinis*
MW380003.1	*Hypostomus francisci*
MW380004.1	*Hypostomus* sp.
MW380005.1	*Hypostomus nigrolineatus*
MW380006.1	*Hypostomus* sp.
MW380007.1	*Hypostomus francisci*
MW380008.1	*Hypostomus alatus*
MW380009.1	*Hypostomus francisci*
MW380010.1	*Hypostomus* aff. *auroguttatus*
MW380011.1	*Hypostomus pusarum*
MW380012.1	*Hypostomus unae*
MW380013.1	*Hypostomus unae*

Accession No.	Taxon
MW380014.1	*Hypostomus auroguttatus*
MW380015.1	*Hypostomus affinis*
MW380016.1	*Hypostomus affinis*
MW380017.1	*Hypostomus jaguar*
MW380018.1	*Hypostomus wuchereri*
MW380019.1	*Hypostomus francisci*
MW380020.1	*Hypostomus francisci*
MW380021.1	*Hypostomus* cf. *macrops*
MW380022.1	*Hypostomus francisci*
MW380023.1	*Hypostomus* aff. *francisci*
MW380024.1	*Hypostomus alatus*
MW380025.1	*Hypostomus alatus*
MW380026.1	*Hypostomus* cf. *macrops*
MW380027.1	*Hypostomus velhochico*
MW380028.1	*Hypostomus velhochico*
MW380029.1	*Hypostomus velhochico*
MW380030.1	*Hypostomus* cf. *garmani*
MW380031.1	*Hypostomus* cf. *garmani*
MW380032.1	*Hypostomus* cf. *garmani*
MW380033.1	*Hypostomus* cf. *garmani*
MW380034.1	*Hypostomus* cf. *garmani*
MW380035.1	*Hypostomus* cf. *garmani*
MW380036.1	*Hypostomus* sp.
MW380037.1	*Hypostomus* sp.
MW380038.1	*Hypostomus* aff. *francisci*

Accession No.	Taxon
MW380039.1	*Hypostomus* aff. *francisci*
MW380040.1	*Hypostomus margaritifer*
MW380041.1	*Hypostomus margaritifer*
MW380042.1	*Hypostomus francisci*
MW380043.1	*Hypostomus* sp.
MW380044.1	*Hypostomus* sp.
MW380045.1	*Hypostomus leucophaeus*
MW380046.1	*Hypostomus leucophaeus*
MW380047.1	*Hypostomus leucophaeus*
MW380048.1	*Hypostomus leucophaeus*
MW380049.1	*Pterygoplichthys chrysostiktos*
MW380050.1	*Pterygoplichthys chrysostiktos*
MW380051.1	*Hypostomus velhochico*
MW380052.1	*Hypostomus velhochico*
MW380053.1	*Hypostomus velhochico*
MW380054.1	*Hypostomus velhochico*
MW380055.1	*Hypostomus margaritifer*
MW380056.1	*Hypostomus margaritifer*
MW380057.1	*Hypostomus* cf. *macrops*
MW380058.1	*Hypostomus* cf. *macrops*
MW380059.1	*Hypostomus* aff. *francisci*
MW380060.1	*Hypostomus* aff. *francisci*
MW380061.1	*Hypostomus* cf. *wuchereri*
MW380062.1	*Hypostomus* aff. *francisci*
MW401674.1	*Pterygoplichthys pardalis*

MOLECULAR ECOLOGY

Accession No.	Taxon
MZ050829.1	*Hypostomus plecostomus*
MZ050926.1	*Hypostomus gymnorhynchus*
MZ050957.1	*Hypostomus plecostomus*
MZ051003.1	*Hypostomus watwata*
MZ051068.1	*Hypostomus watwata*
MZ051282.1	*Hypostomus gymnorhynchus*
MZ051559.1	*Hypostomus plecostomus*
MZ051637.1	*Hypostomus plecostomus*
MZ051739.1	*Hypostomus gymnorhynchus*
MZ051754.1	*Hypostomus plecostomus*
MZ051871.1	*Hypostomus gymnorhynchus*
MZ051917.1	*Hypostomus gymnorhynchus*
MZ051945.1	*Hypostomus plecostomus*
MZ052001.1	*Hypostomus gymnorhynchus*
MZ345687.1	*Hypostomus annectens*
MZ345689.1	*Hypostomus* sp.
MZ345690.1	*Hypostomus* sp.
MZ345691.1	*Hypostomus* sp.
MZ351897.1	*Hypostomus pusarum*
MZ351898.1	*Hypostomus pusarum*
MZ351899.1	*Hypostomus pusarum*
MZ351900.1	*Hypostomus pusarum*
MZ351901.1	*Hypostomus sertanejo*
MZ351902.1	*Hypostomus sertanejo*
MZ351903.1	*Hypostomus johnii*

Accession No.	Taxon
MZ351904.1	*Hypostomus johnii*
MZ351905.1	*Hypostomus* sp.
MZ351906.1	*Hypostomus* sp.
MZ351907.1	*Hypostomus* sp.
MZ351908.1	*Hypostomus* sp.
MZ351909.1	*Hypostomus* sp.
MZ351910.1	*Hypostomus* sp.
MZ351911.1	*Hypostomus vaillanti*
MZ351912.1	*Hypostomus vaillanti*
MZ351913.1	*Hypostomus vaillanti*
MZ351914.1	*Hypostomus vaillanti*
MZ351915.1	*Hypostomus* cf. *pusarum*
MZ351916.1	*Hypostomus* cf. *pusarum*
MZ351917.1	*Hypostomus* cf. *pusarum*
MZ351918.1	*Hypostomus* cf. *pusarum*
MZ351919.1	*Hypostomus* cf. *pusarum*
MZ351920.1	*Hypostomus velhochico*
MZ351921.1	*Hypostomus velhochico*
MZ351922.1	*Hypostomus velhochico*
MZ351923.1	*Parotocinclus spilurus*
NC_049098.1	*Corydoras trilineatus*
NC_063780.1	*Corydoras aeneus*
NC_063781.1	*Corydoras paleatus*
NC_072072.1	*Corydoras hastatus*
NC_077553.1	*Corydoras julii*

MOLECULAR ECOLOGY

Accession No.	Taxon
NC_015747.1	*Pterygoplichthys disjunctivus*
OQ200385.1	*Hypostomus sertanejo*
OQ200386.1	*Hypostomus sertanejo*
OQ200387.1	*Hypostomus pusarum*
OQ200388.1	*Hypostomus pusarum*
OQ200389.1	*Hypostomus pusarum*
OQ200390.1	*Hypostomus pusarum*
OQ200391.1	*Hypostomus pusarum*
OQ200392.1	*Hypostomus pusarum*
OQ200393.1	*Hypostomus pusarum*
OQ200394.1	*Hypostomus pusarum*
OQ200395.1	*Hypostomus pusarum*
OQ200396.1	*Hypostomus pusarum*
OQ200397.1	*Hypostomus pusarum*
OQ200398.1	*Hypostomus pusarum*
OQ200399.1	*Hypostomus pusarum*
OQ200400.1	*Hypostomus pusarum*
OQ200401.1	*Hypostomus pusarum*
OQ200402.1	*Hypostomus pusarum*
OQ200403.1	*Hypostomus pusarum*
OQ200404.1	*Hypostomus pusarum*
OQ200405.1	*Hypostomus pusarum*
OQ200406.1	*Hypostomus pusarum*
OQ200407.1	*Hypostomus* sp.
OQ200408.1	*Hypostomus* sp.

MOLECULAR ECOLOGY

Accession No.	Taxon
OQ200409.1	*Hypostomus* sp.
OQ200410.1	*Hypostomus* sp.
OQ200411.1	*Hypostomus* sp.
OQ200412.1	*Hypostomus* sp.
OQ200413.1	*Hypostomus* sp.
OQ200414.1	*Hypostomus* sp.
OQ200415.1	*Hypostomus* sp.
OQ200416.1	*Hypostomus* sp.
OQ200417.1	*Hypostomus* sp.
OQ200418.1	*Hypostomus pusarum*
OQ231490.1	*Hypostomus plecostomus*
OQ231491.1	*Hypostomus plecostomus*
OQ231492.1	*Hypostomus plecostomus*
OR732968.1	*Hypostomus cochliodon*
PP998994.1	*Hypostomus khimaera*
PP998995.1	*Hypostomus khimaera*
PP998996.1	*Hypostomus khimaera*
PP998997.1	*Hypostomus khimaera*
PP998998.1	*Hypostomus khimaera*
PP998999.1	*Hypostomus khimaera*
PP999000.1	*Hypostomus khimaera*
PQ233434.1	*Hypostomus* sp.
PQ233441.1	*Hypostomus hondae*
PQ233442.1	*Hypostomus hondae*
PQ233580.1	*Hypostomus* sp.

MOLECULAR ECOLOGY

Accession No.	Taxon
PQ233585.1	*Hypostomus* sp.
PQ233596.1	*Hypostomus hondae*
PQ233603.1	*Hypostomus* sp.
PQ233605.1	*Hypostomus* sp.
PQ233607.1	*Hypostomus* sp.
PQ233660.1	*Hypostomus hondae*
PQ233702.1	*Hypostomus hondae*
PQ233846.1	*Hypostomus hondae*
PV077306.1	*Hypostomus ancistroides*
PV077307.1	*Hypostomus ancistroides*
PV077308.1	*Hypostomus ancistroides*
PV077309.1	*Hypostomus ancistroides*
PV077310.1	*Hypostomus ancistroides*
PV077311.1	*Hypostomus ancistroides*
PV077312.1	*Hypostomus ancistroides*
PV077313.1	*Hypostomus ancistroides*
PV077314.1	*Hypostomus ancistroides*
PV077315.1	*Hypostomus ancistroides*
PV077316.1	*Hypostomus ancistroides*
PV077317.1	*Hypostomus ancistroides*
PV077318.1	*Hypostomus ancistroides*
PV241661.1	*Hypostomus plecostomus*
PV241662.1	*Hypostomus plecostomus*

MOLECULAR ECOLOGY

Supplementary Table 9: Cross-validation error rate for K = 1 through K = 3 for all-population and "true" *Hypostomus*-only ADMIXTURE analyses. The lowest value indicates the optimal number of clusters representing genetic variation in the samples.

ADMIXTURE analysis	Cross-validation (CV) error
**All-population**	
K=1	0.94301
K=2	0.46520
K=3	0.42119
K=4	0.44448
K=5	0.58914
**San Marcos River and San Felipe Creek populations only**	
K=1	0.62623
K=2	0.56252
K=3	0.64117
K=4	0.70639
K=5	0.78484

MOLECULAR ECOLOGY

Supplementary Figure 1: IQ-TREE2 analysis showing a phylogenetic hypothesis of the mitochondrial gene cytochrome c oxidase I (COI). Samples from the San Marcos River, San Felipe Creek, and Comal River in our study are highlighted in green.

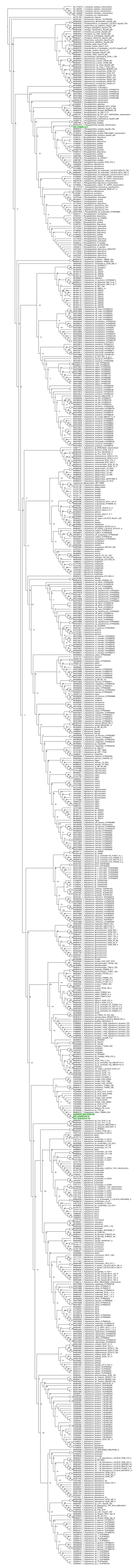

MOLECULAR ECOLOGY

Supplementary Figure 2: ADMIXTURE results (K = 1–5) showing genetic population structure of suckermouth armored catfish in (a) all three Texas rivers and (b) only “true” Hypostomus species, indicating evidence of historical gene flow.

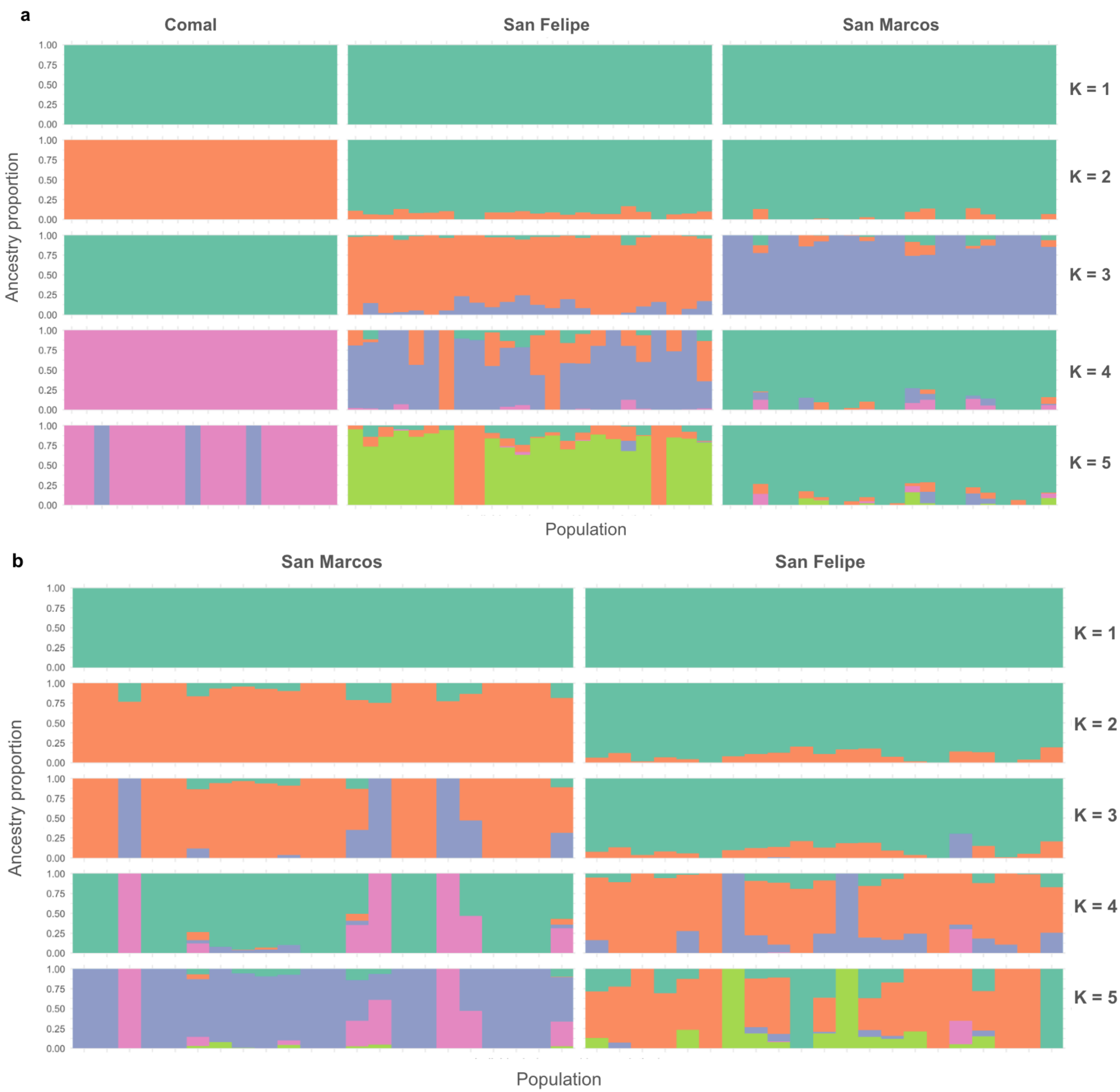

MOLECULAR ECOLOGY

Supplementary Figure 3: Principal component analysis (PCA) plot of “true” *Hypostomus* samples aligned to the *Hypostomus* reference genome. Shapes indicate sex, with triangles representing females and circles representing males.

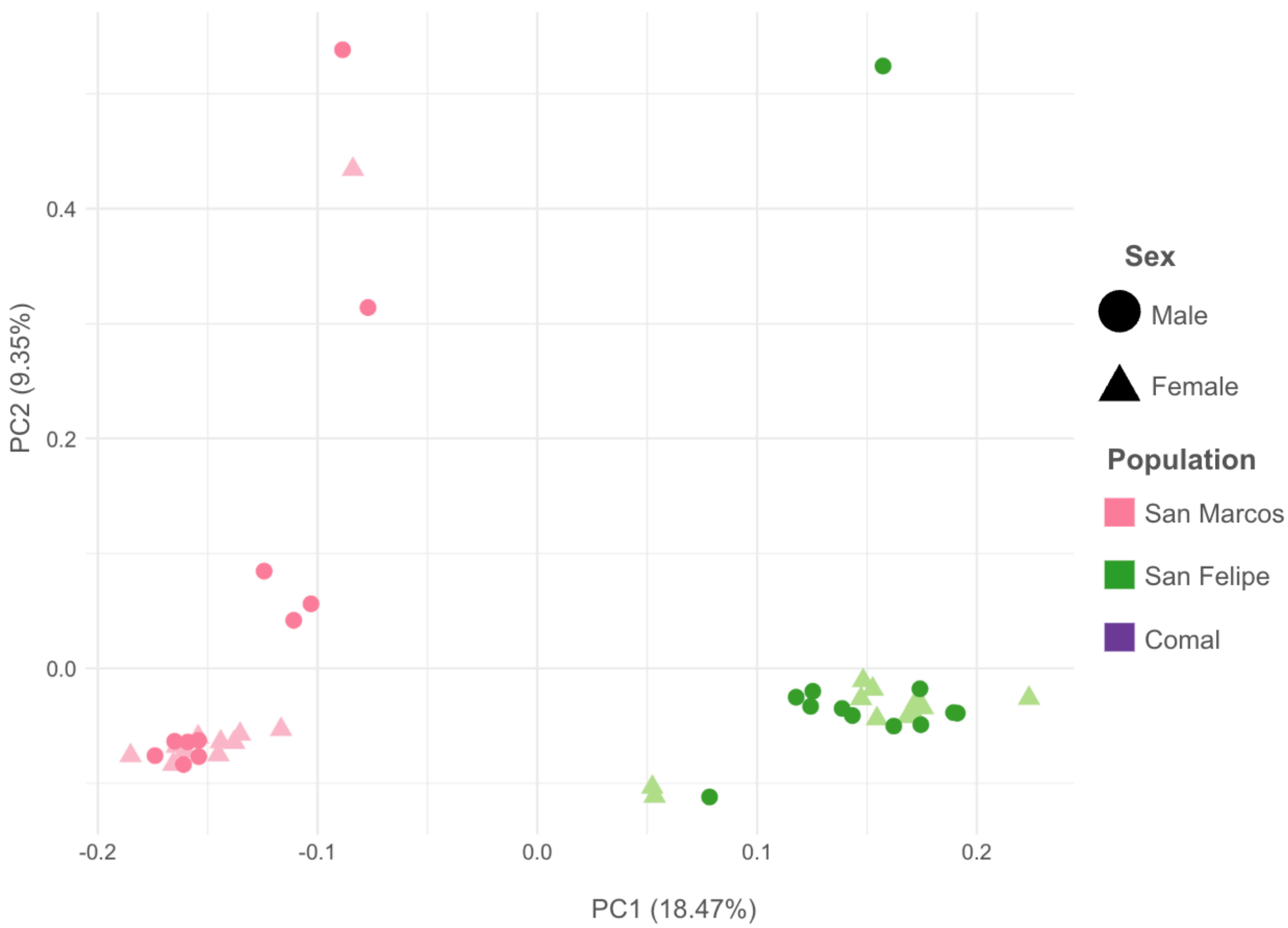

MOLECULAR ECOLOGY

Supplementary Figure 4: Top 30 scaffolds in *Pterygoplichthys* genome with the highest density of sex-specific k-mers in 50kb windows and corresponding coverage of the opposite sex (a, b). Axes have been made the same between comparison pairs, and the scale of the density plot is similar to the *Hypostomus* spp., to emphasize differences, which highlighted the fact that the Comal population has an overall lower density of sex-specific k-mers.

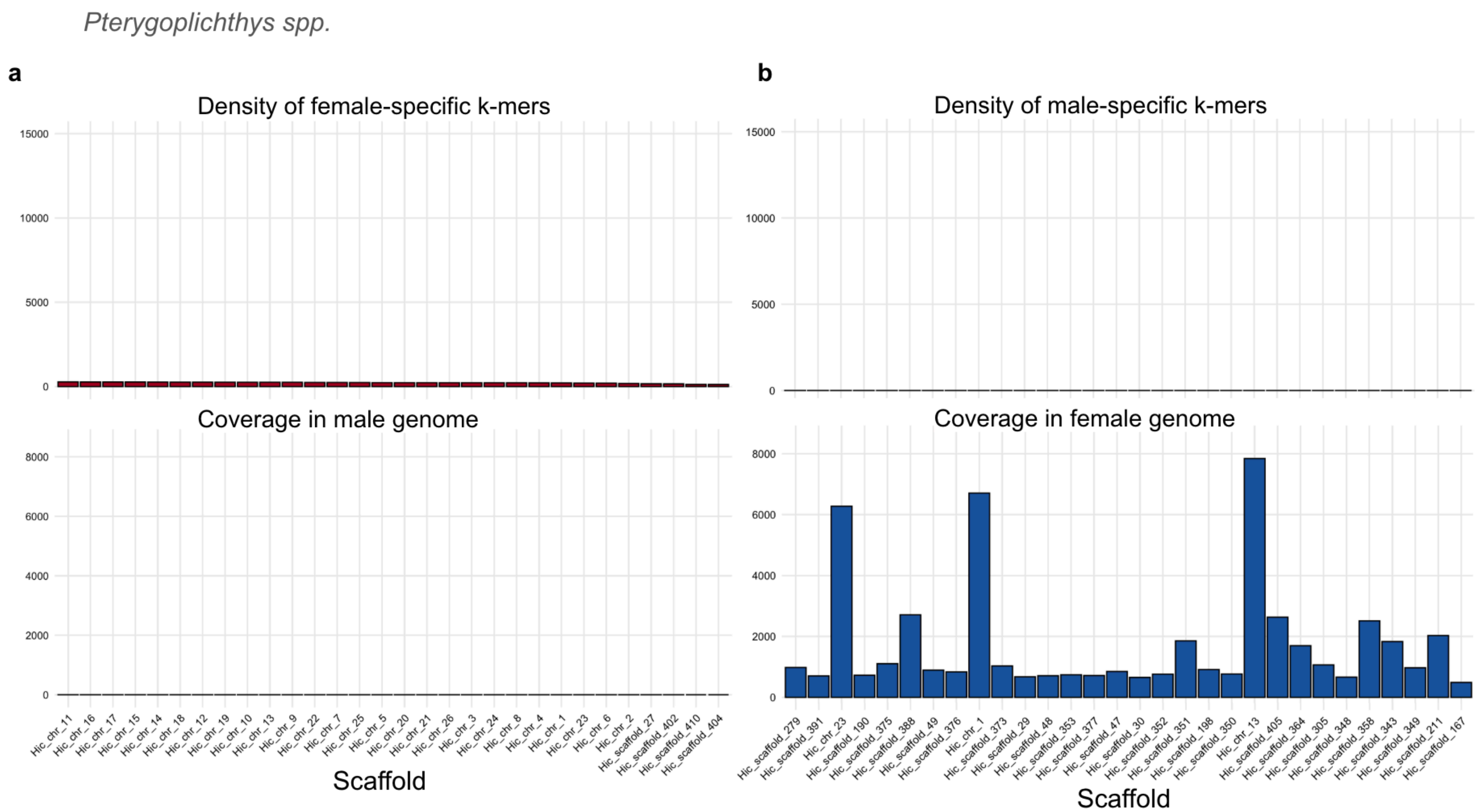

Supplement: Supplementary file 1 [file MEC-35-e70609-s001.pdf]
